# Saxifragifolin D attenuates phagosome maturation arrest in *Mycobacterium tuberculosis*-infected macrophages via an AMPK and VPS34-dependent pathway

**DOI:** 10.1186/s13568-016-0317-6

**Published:** 2017-01-03

**Authors:** Jia Zhou, Rui Xu, Xian-zhi Du, Xiang-dong Zhou, Qi Li

**Affiliations:** 1 Department of Respiratory Medicine, The First Affiliated Hospital of Chongqing Medical University, Yu-zhong District, Chongqing, 400016 China; 2 Department of Respiratory Medicine, The Second Affiliated Hospital, Chongqing Medical University, No.74 Linjiang Road, Yu-zhong District, Chongqing, 400010 China; 3 Department of Respiratory Medicine, the First Affiliated Hospital of Hainan Medical University, Haikou, Hainan province 570102 China

**Keywords:** Saxifragifolin D, Phagosome maturation, *Mycobacterium tuberculosis*, VPS34

## Abstract

Saxifragifolin D (SD), a traditional Chinese medicine, is a pentacyclic triterpenoid compound first isolated from *Androsace umbellata*. Various plant triterpenoids have been reported to exhibit antitubercular activity. In this study, THP-1-derived macrophages were infected with an attenuated *M. tuberculosis* (*M.tb*) strain, H_37_Ra. Intracellular replication of *M.tb* was evaluated by counting the colonies after 4 weeks of incubation. The results indicated that SD treatment reduced the intracellular replication of *M.tb* in THP-1-derived macrophages but not in A549 cells. We performed a phagosome maturation test using confocal microscopy and found that SD treatment partially attenuated the phagosome arrest induced by *M.tb* infection. These effects were dependent on a VPS34-associated pathway. Immunoprecipitation assays showed that SD increased intracellular UVRAG-linked VPS34, the active VPS34 complex II. However, SD had no effect on the total VPS34 pool. Moreover, the results indicated that the SD-mediated increase in VPS34 complex II activity was mediated by an AMPK-dependent pathway. Collectively, these data indicate that SD may be a promising candidate for treatment of *M.tb*.

## Introduction

Tuberculosis(TB) continues to be a major threat to human health, with an estimated global mortality of 1.43 million people each year (Maxmen 2013). The only approved vaccine, Bacille–Calmette–Guerin (BCG), an attenuated strain of *Mycobacterium bovis*, has been shown to protect children from severe TB but fails to block the transmission chain in adults. *Mycobacterium tuberculosis* (*M.tb*), is the causative agent of TB and can survive and replicate in macrophages. *M.tb* virulence is multifarious but initially relies on its ability to parasitize phagocytic cells and escape from the host immune system (Flannagan et al. 2012). The major function of alveolar macrophages is uptake of inhaled microorganisms through phagocytosis. This event depends on the formation of the phagosome, which then matures into a phagolysosome through a series of fusion reactions with lysosomes (Flannagan et al. 2012). In contrast to primary phagosomes, mature phagolysosomes contain antimicrobial peptides and hydrolases that are predominantly active under an acidic pH. Therefore, the maturation of the phagosome is crucial for alveolar macrophages to kill the captured microbes. *M.tb* is inhaled as an aerosol and phagocytosed by alveolar macrophages. Forty years ago, investigators reported that inhibition of phagosome maturation was the major virulence mechanism of *M.tb*. Since then, numerous mechanisms underlying the disruption of phagosome maturation have been identified in *M.tb* infection (Russell 2011; Russell et al. 2013). Early work showed that *M.tb* predominantly blocked phagosome maturation by two small GTPases, Rab5 and Rab7, in membrane trafficking and fusion on early and late endosomes (Via et al. 1997). EEA1, the effector of Rab5, facilitates the delivery of Cathepsin D and the H^+^-ATPase subunit Vo from the trans-Golgi-network to the phagosome. This step is essential in phagosome maturation and acidification (Fratti et al. 2003b). Later experiments revealed that the specific mechanism of phagosome maturation arrest is principally dependent on impairment of EEA1 recruitment, which is mediated by Rab5 and phosphatidylinositol 3-phosphate (PI3P) (Fratti et al. 2001). *M.tb* inhibits PIP3 production, which is responsible for the acquisition of lysosomal characteristics (Chua and Deretic 2004). Recent studies identified several components of *M.tb*, such as ManLAM, which block phagosome maturation by reducing PI3P production via inhibition of VPS34, a unique class III PI3K (Purdy et al. 2005).

Although treatment of drug-susceptible *M.tb* is effective, drug toxicity, reduced discovery of new drugs and increasing multi-drug resistance of *M.tb* indicate that new therapeutic strategies are needed (Zumla et al. 2013). As conventional pathogen-targeted strategies have the disadvantage of inducing microbial resistance, a new approach might focus on the modulation of host cellular immunity to improve pathogen eradication (Ejim et al. 2011). Recently, researchers reported that AMPK activators controlled the intracellular growth of *M.tb* in macrophages. To date, studies have shown that the AMPK-associated signalling pathway is the major activator of VPS34 (Xu et al. 2016; Yang et al. 2014).

Saxifragifolin D (SD), a pentacyclic triterpenoid compound, was first isolated from *Androsace umbellate* and is commonly used in the treatment of solid tumours in Chinese traditional medicine (Park et al. 2010). Based on evidence showing that pentacyclic triterpenoids are novel activators of AMPK and may indirectly up-regulate VPS34 (Akhtar et al. 2016; Shi et al. 2013), we determined whether the active ingredients of the Chinese herbal medicine SD could inhibit the intracellular growth of *M.tb* and promote the maturation of phagosomes in macrophages.

## Materials and methods

### Reagents and chemicals

SD (purity >95%) was isolated from *A. umbellate* and dissolved in dimethyl sulfoxide (DMSO) at a concentration of 20 mM (Wang et al. 2008). The attenuated *M.tb* strain (H37Ra) was purchased from China National Institution For bio-product and drag control. Human monocytic THP-1 cells and A549 cells were purchased from the American Type Culture Collection (Manassas, VA, USA) and maintained in RPMI1640 containing 10% FBS and 1% penicillin/streptomycin mix. Rabbit anti-human VPS34 antibody (ab5451), rabbit anti-human UVRAG antibody (ab174550), rabbit anti-human AMPK antibody (ab131512) and phosphorylated AMPK (ab72845) antibodies were purchased from Abcam (Cambridge, MA, USA). The internal reference beta-actin antibody and secondary antibodies, including horseradish peroxidase (HRP)-conjugated goat anti-rabbit IgG, were purchased from Zhongshan Goldenbridge Biotechnology (Peking, China).

### Cell culture

The monocytic cell line THP-1 or A549 was maintained in RPMI 1640 supplemented with 10% foetal calf serum, 2 mM l-glutamine, 100 μg/ml streptomycin and 100 U/ml penicillin. Cells were cultured in an incubator at 37 °C with 5% CO_2_. Before treatments, THP-1 cells were seeded in 6-well culture dishes at a density of approximately 2 × 10^6^/ml in fresh medium. THP-1 cells were then stimulated with 20 ng/ml phorbol 12-myristate 13-acetate (PMA, Sigma-Aldrich) for 1 day to induce macrophage-like cells.

### Cell infection and bacterial enumeration

The attenuated *M.tb* strain (H_37_Ra) were used and cultured in Proskauer and Beck (P and B) medium supplemented with 0.05% Tween-80. For visualization of the phagocytosis of *M.tb* by THP-1 cells, 1 ml of H_37_Ra bacterial suspension (10^9^/ml) was labelled by incubation with 0.5 mg FITC (Sigma) in 0.1 M carbonate buffer (pH 9.0) at 37 °C for 2 h (Stokes et al. 1993). The bacterial suspension was centrifuged and washed with PBS 3 times to remove unbound FITC. Before infection, the cells were cultured in 6-well plates and washed with RPMI1640 3 times to remove serum and antibiotics. H_37_Ra bacteria were re-suspended and diluted in antibiotic-free RPMI1640 to infect THP-1-derived macrophages or A549 cells with a multiplicity of infection (MOI) of 5. The infected cells were maintained in an incubator at 37 °C with 5% CO_2_ for 2 h. The cells were then washed with PBS 3 times to remove the extracellular bacteria. Every 48 h, the culture medium was replaced.

On days 0, 2, 4 and 6, the infected THP-1 or A549cells were lysed with1 ml 0.025% SDS to release the intracellular *M.tb*. The lysate were then plated as serial dilutions on MB7H11 agar. Colonies were counted after 4 weeks of incubation at 37 °C, and the data are expressed as CFU/ml.

### Western blot analysis

The expression of the VPS34, UVRAG, AMPK and phosphorylated AMPK protein in each treatment group was detected by western blotting. Briefly cells were washed with ice-cold PBS 3 times and lysed on ice for 20 min with a western cell lysis buffer (Beyotime, Shanghai, China) containing PMSF, protease inhibitors, and phosphatase inhibitors. The lysis products were then centrifuged at 12,000 rpm for 15 min at 4 °C. Supernatants were standardized for equal protein concentration following the instructions of the BCA Protein Assay Kit (Beyotime, Shanghai, China). The samples were then mixed with loading buffer and boiled in water for 10 min. After separation by sodium dodecyl sulphate polyacrylamide gel electrophoresis (SDS-PAGE), the proteins were transferred onto polyvinylidene difluoride (PVDF) membranes. The PVDF membranes were blocked in 5% (w/v) non-fat dried skim milk powder or 5% BSA (for detecting phosphorylated AMPK)and incubated with diluted primary antibody (1:300 for VPS34, 1:1000 for UVRAG, 1:800 for AMPK, 1:300 for phosphorylated AMPK, 1:200 for beta-actin) overnight at room temperature. After the membranes were washed with PBST 3 times for 15 min, they were incubated with the secondary antibody, goat anti-rabbit IgG (HRP), at a 1:3000 dilution for 2 h. Blots were visualized using enhanced chemiluminescence following the protocol of the manufacturer of the reagent kit (KeyGen, Nanjing, China). The intensity of each band was measured using the Fluor-S MultiImager and QuantityOne software (Bio-Rad, Hercules, CA, USA).

### Co-immunoprecipitation (Co-IP)

Co-IP was performed following the instructions of the Co-Immunoprecipitation Kit (Thermo Scientific) (Peking, China). Generally, the cells were washed with PBS 3 times and lysed on ice for 30 min using IP lysis buffer (Beyotime) (Beijing, China) containing protease inhibitors (Thermo Scientific) (Peking, China). For removal of the nuclei and intact cells, the lysis products were centrifuged at 20,000×*g* for 15 min at 4 °C. Protein A agarose was washed and diluted by PBS. The diluted Protein A agarose (50%) was added to the protein at a 1:10 (v/v) ratio. The mixture was incubated on a 4 °C shaking table for 30 min and then centrifuged at 20,000×*g* for 15 min at 4 °C to collect the supernatant. The supernatants were standardised at 5 μg/μl for equal protein concentration following the instructions in the Bicinchoninic Acid Protein Assay kit (Beyotime, Peking, China). Then, 5 μg rabbit anti-human VPS34 was added to an Eppendorf tube containing 200 μl protein and incubated on a 4 °C shaking table overnight. The antigen–antibody complexes were captured by adding 100 μl Protein A agarose to the samples for 90 min at room temperature. After centrifugation at 20,000×*g* for 1 min, the Protein A agarose containing the antigen–antibody complexes was washed with ice-cold PBS 3 times. The precipitates were mixed with 5× western blot loading buffer and boiled for 5 min. After centrifugation at 20,000×*g* for 15 min, the supernatants were prepared for SDS-PAGE.

### Analysis of phagosome maturation by confocal microscopy

THP-1-derived macrophages were plated at a density of 2 × 10^5^/ml in 12-well plates on a poly-l-lysine coated glass coverslip in each well. THP-1-derived macrophages were washed 3 times by PBS and infected with 1 × 10^6^/ml FITC-labelled *M.tb* for 2 h at 37 °C. Cells were then washed 3 times with RPMI1640 to remove extracellular bacteria. After 6 days of culture at 37 °C, the cells were incubated for 20 min in RPMI1640 containing 70 nM LysoTracker Deep Red (ThermoFisher Scientific, ShangHai, China). Then, THP-1-derived macrophages were fixed with 4% paraformaldehyde for 15 min. Slides were then visualized using a confocal microscope (TCS-SP5, Leica, Germany). Co-localization was determined by identifying FITC-linked *M.tb* (Green) with over lapping LysoTracker(Red). Representative images were obtained with a digital camera and were then processed using Adobe Photoshop 7.0.

## Results

### Cell viability assay

**Fig. 1:**
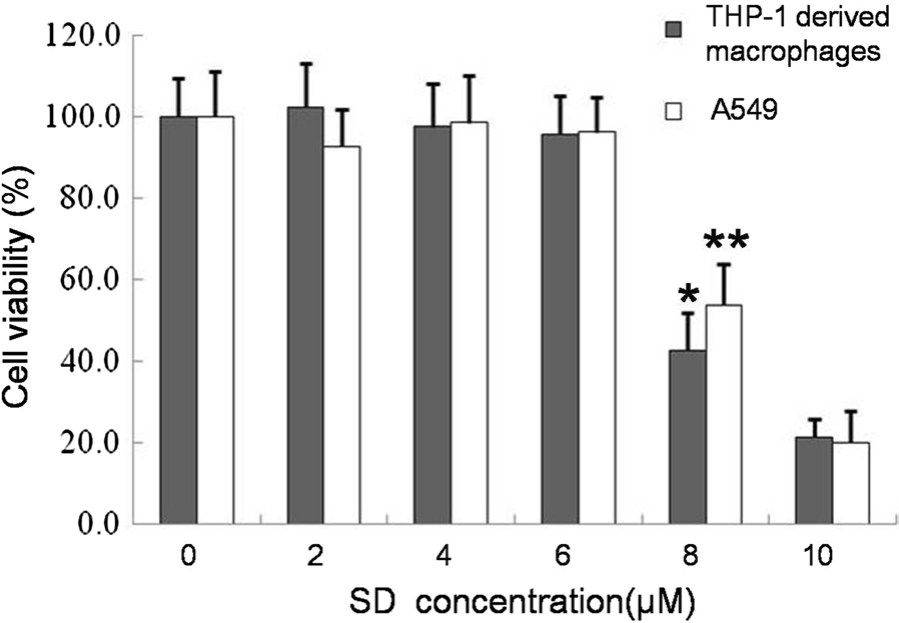
MTT assays for cell viability. THP-1-derived macrophages or A549 cells propagated in RPMI 1640 culture medium were used as negative controls. Cells were exposed to different concentrations of SD. Data are represented as the mean ± SD; n = 4, *P < 0.05 versus THP-1controls; **P < 0.05 versus A549 controls

THP-1-derived macrophages or A549 cells were seeded in 96-well plates and exposed to different concentrations of SD (ranging from 2 to 10 μM) for 6 days. Cell viability was assessed by MTT assays. The result demonstrated a significant reduction of cell viability in cells cultured in 8 μM SD for 6 days from both cell lines compared to that of the controls (Fig. 1).

### SD decreases the intracellular replication of *M.tb* inTHP-1-derived macrophages but not A549 cells

**Fig. 2:**
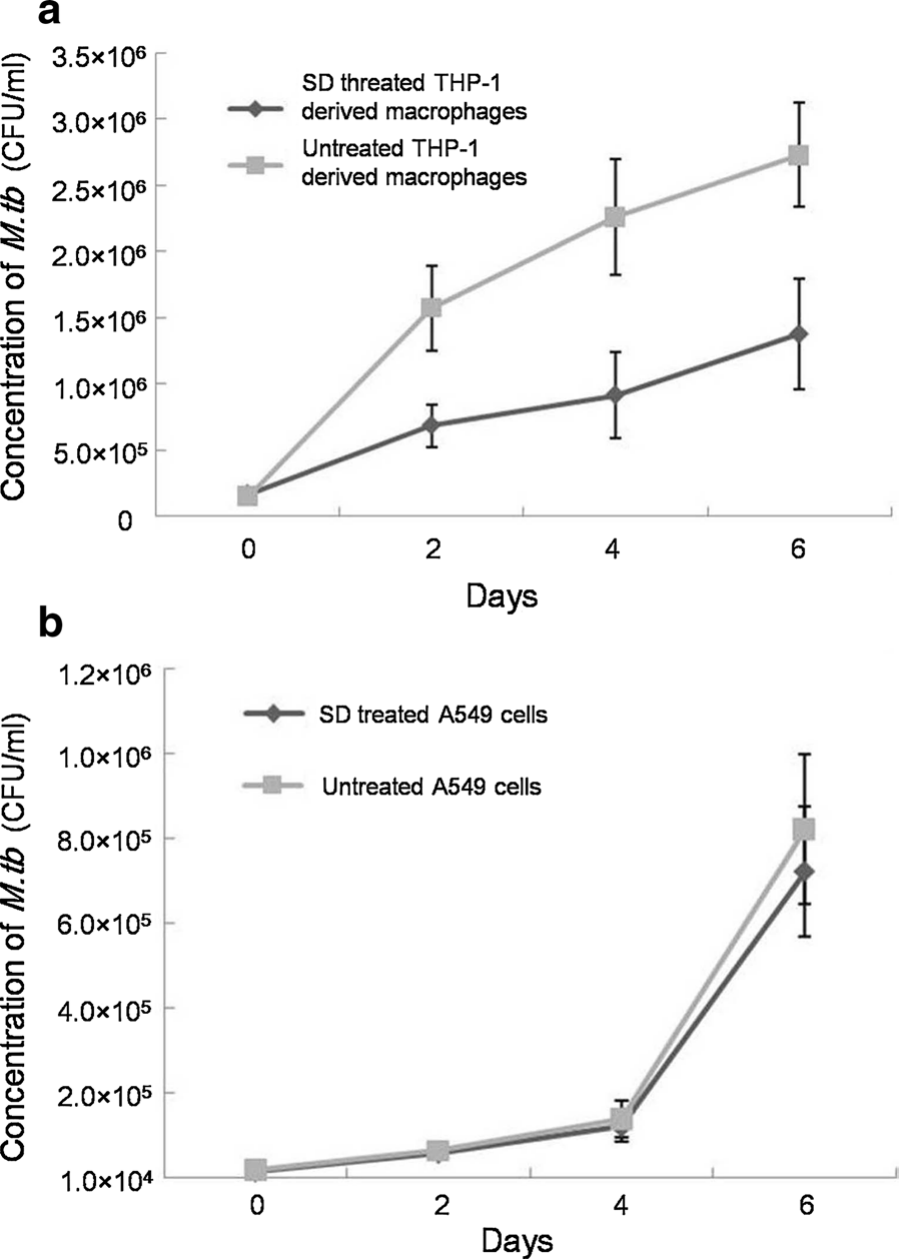
**G**rowth of *M.tb* in THP-1-derived macrophages (**a**) and A549 cells (**b**). Results are expressed as CFU/ml. The data represent the average and the standard deviation obtained from 6 parallel independent infections. The experiment was repeated 4 times

Intracellular growth of *M.tb* was evaluated by counting the colonies after 4 weeks of incubation at 37 °C. A significant difference was observed in the growth of *M.tb* in THP-1-derived macrophages treated by SD (6 μM) compared to that of the untreated THP-1-derived macrophages. Forty-eight hours post-infection, the growth of intracellular *M.tb* was significantly inhibited by SD treatment compared to that of the negative control. Six days post-infection, the growth of intracellular *M.tb* in SD-treated THP-1-derived macrophages was only approximately half that of the control THP-1 cells (Fig. 2a). Given that *M.tb* can also infect alveolar epithelial cells, we performed similar investigations using A549 human type II alveolar epithelial cells. However, SD treatment did not affect the intracellular replication of *M.tb* in A549 cells (Fig. 2b).

### SD increases phagosomal maturation in THP-1-derived macrophages during *M.tb* infection

**Fig. 3:**
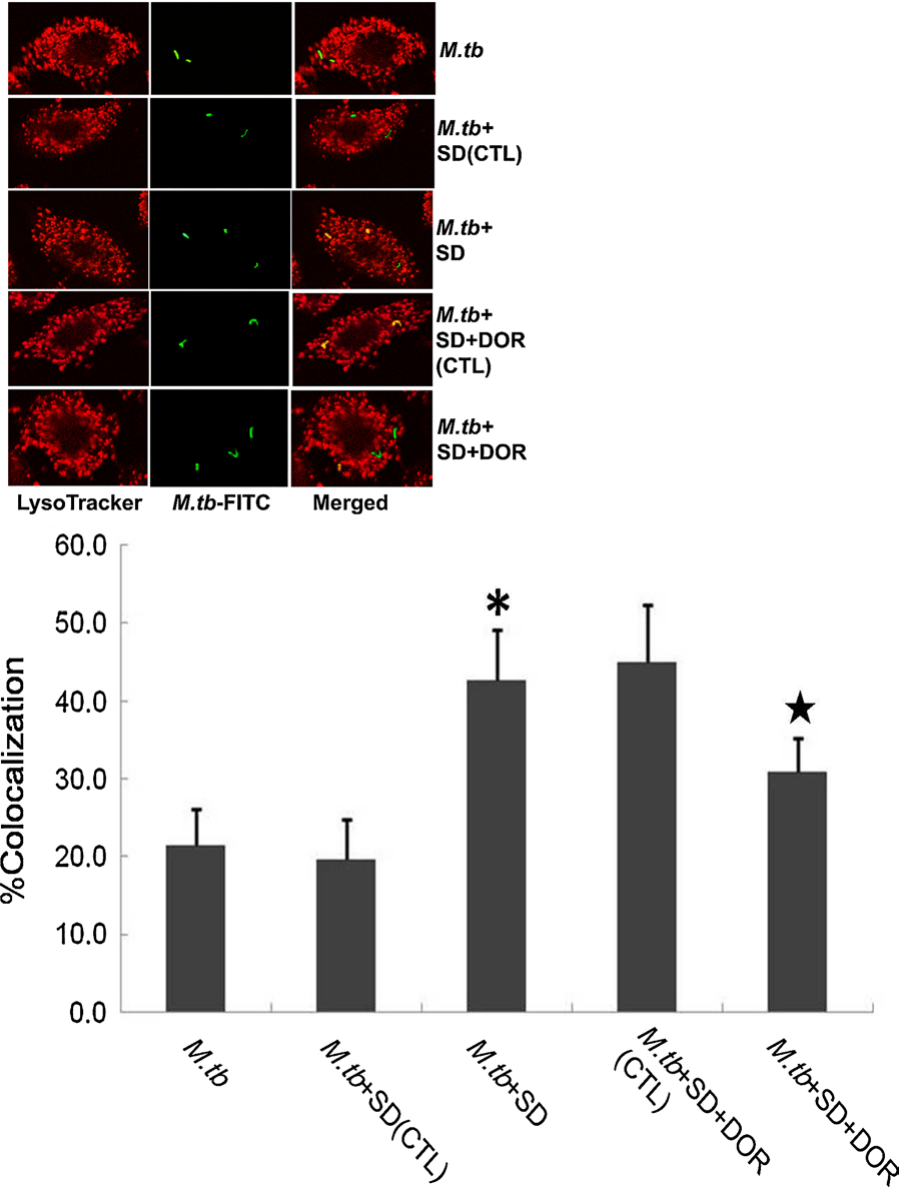
Confocal microscopy analysis of the co-localization of *M.tb* and lysosomes. THP-1-derived macrophages were infected with FITC-labelled *M.tb* (*green*) at an MOI of 5. After 6 days of culture at 37 °C, the cells were treated with LysoTracker (*red*) for 20 min. The results are expressed as the percentage of co-localization of *M.tb* and lysosome. (1) *M.tb*, *M.tb*-infected macrophages were maintained in RPMI1640 culture medium for 6 days; (2) *M.tb*+SD(CTL), *M.tb*-infected macrophages were maintained in RPMI1640 culture medium containing DMSO (the solution medium of SD) for 6 days. DMSO was added to the culture medium at the same volume of SD; (3) *M.tb*+SD, *M.tb*-infected macrophages were maintained in RPMI1640 culture medium containing 6 μM SD for 6 days; (4) *M.tb*+SD+DOR(CTL), *M.tb*-infected macrophages were maintained in RPMI1640 culture medium containing 6 μM SD and the solution medium of dorsomorphin (DOR), DMSO, for 6 days. DMSO was added to the culture medium based on the volume of DOR; (5) *M.tb*+SD+DOR, *M.tb*-infected macrophages were maintained in RPMI1640 culture medium containing 6 μM SD and 1 μM DOR for 6 days. Data are shown as the mean ± S.D. of 4 independent experiments carried out in triplicate, with a minimum of 50 phagosomes counted per experiment for each sample. *P < 0.05 versus *M.tb* group; ^★^P < 0.05 versus *M.tb*+SD group.* Colour* figure online only

Because SD could decrease *M.tb* replication in macrophages, we next investigated the involvement of SD in phagosomal maturation by studying the co-localization of the FITC-labelled *M.tb* strain H_37_Ra with LysoTracker Red. We observed that only (21.4 ± 4.7)% of FITC-labelled *M.tb* co-localized with LysoTracker-rich compartments in untreated THP-1-derived macrophages. However, significantly more co-localization of *M.tb* and LysoTracker-rich compartments, (42.7 ± 6.5)%, was found in SD-treated THP-1 derived macrophages (Fig. 3).

### SD increases the proportion of the VPS34/VPS15/Beclin-1/UVRAG tetramer in the VPS34 total pool in an AMPK-dependent manner during *M.tb* infection

**Fig. 4:**
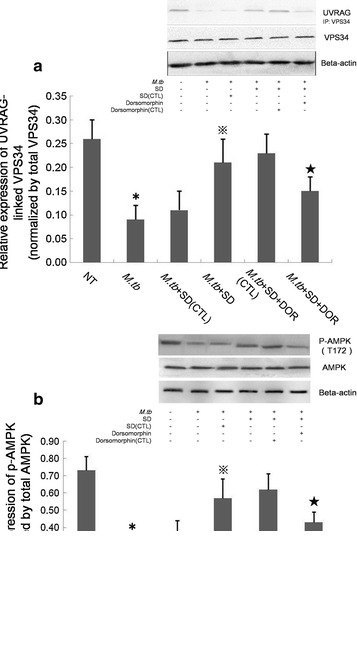
Western blot analysis of VPS34 levels in THP-1-derived macrophages. **a** UVRAG-linked VPS34 was detected by co-IP. The results are shown as the relative UVRAG-linked VPS34 normalized to total VPS34. **b** Phosphorylated AMPK was detected by western blot. Data are shown as the relative p-AMPK levels normalized to total AMPK. (1) NT, THP-1-derived macrophages were maintained in RPMI1640 culture medium for 6 days; (2) *M.tb*, *M.tb*-infected macrophages were maintained in RPMI1640 culture medium for 6 days; (3) *M.tb*+SD(CTL), *M.tb*-infected macrophages were maintained in RPMI1640 culture medium containing DMSO(the solution medium of SD) for 6 days. DMSO was added to the culture medium at the same volume as SD; (4) *M.tb*+SD, *M.tb*-infected macrophages were maintained in RPMI1640 culture medium containing 6 μM SD for 6 days; (5) *M.tb*+SD+DOR(CTL), *M.tb*-infected macrophages were maintained in RPMI1640 culture medium containing 6 μM SD and the solution medium of DOR(DMSO) for 6 days. The solute medium of DOR was added to the culture medium based on the volume of DOR; (6) *M.tb*+SD+DOR, *M.tb*-infected macrophages were maintained in RPMI1640 culture medium containing 6 μM SD and 1 μM DOR for 6 days. Data are shown as the mean ± S.D. of 4 independent experiments carried out in triplicate. *P < 0.05 versus NT group; ^※^P < 0.05 versus *M.tb* group; ^★^P < 0.05 versus *M.tb*+SD group

Because SD was a novel activator of AMPK and may indirectly activate VPS34, we assessed the formation of the VPS34/VPS15/Beclin-1/UVRAG tetramer, which is the active form in the VPS34 pool. As expected, *M.tb* infection did not influence the synthesis of VPS34 but decreased the UVRAG-linked VPS34 in THP-1-derived macrophages (Fig. 4a). SD treatments increased the UVRAG-linked VPS34 tetramers in *M.tb*-infected macrophages but not total VPS34, indicating that SD did not influence the total VPS34 pool in macrophages.

**Fig. 5:**
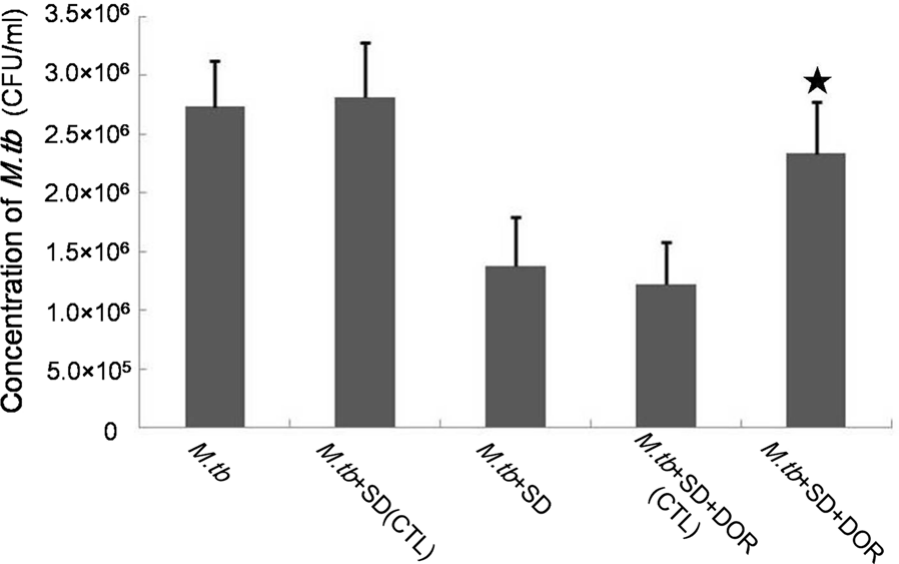
Growth of *M.tb* in THP-1-derived macrophages. Results are expressed as CFU/ml. (1) *M.tb*, *M.tb*-infected macrophages were maintained in RPMI1640 culture medium for 6 days; (2) *M.tb*+SD(CTL), *M.tb*-infected macrophages were maintained in RPMI1640 culture medium containing DMSO (the solution medium of SD) for 6 days. DMSO was added to the culture medium at the same volume as SD; (3) *M.tb*+SD, *M.tb*-infected macrophages were maintained in RPMI1640 culture medium containing 6 μM SD for 6 days; (4) *M.tb*+SD+DOR(CTL), *M.tb*-infected macrophages were maintained in RPMI1640 culture medium containing 6 μM SD and the solution medium of DOR(DMSO) for 6 days. The solute medium of DOR was added to the culture medium based on the volume of DOR; (5) *M.tb*+SD+DOR, *M.tb*-infected macrophages were maintained in RPMI1640 culture medium containing 6 μM SD and 1 μM DOR for 6 days. Data are shown as the mean ± SD of 4 independent experiments carried out from 6 parallel infections. ^★^P < 0.05 versus *M.tb* + SD group

To analyse the involvement of AMPK in the activation of VPS34 induced by SD, we further examined phosphorylated AMPK in *M.tb*-infected macrophages. Phosphorylated AMPK was significantly decreased by *M.tb* infection. In contrast, SD augmented AMPK phosphorylation in *M.tb*-infected macrophages. An AMPK specific inhibitor, dorsomorphin(DOR), could partially attenuate the up-regulation of p-AMPK induced by pre-treatment of SD, indicating that an AMPK-dependent signalling pathway is involved in SD-induced VPS34 activation in *M.tb*-infected THP-1-derived macrophages (Fig. 4b). To determine whether AMPK signalling was linked to enhanced phagosomal maturation following SD treatment, we performed fluorescence microscopy analyses. After 6 days of infection, *M.tb was* demonstrated (42.7 ± 6.5)% co-localization with LysoTracker Red in SD-treated THP-1-derived macrophages, whereas *M.tb* was showed significantly less co-localization (30.9 ± 4.4%) with LysoTracker Red after DOR pretreatment (Fig. 3). To demonstrate the involvement of AMPK signalling in the inhibition of *M.tb* replication in THP-1-derived macrophages following SD treatment, we counted the *M.tb* colonies after 4 weeks of incubation at 37 °C. The intracellular replication of *M.tb* was demonstrated approximate 1.7-fold higher in *M.tb*+SD+DOR group than that in *M.tb*+SD group according to the *M.tb* colonies after 4 weeks of incubation at 37 °C (Fig. 5).

## Discussion

A key factor of *M.tb* pathogenesis is its ability to survive in host macrophages. Phagosomes mature into phagolysosomes, as evidenced by a decrease in pH and increased activity of hydrolytic enzymes. However, the maturation of *M.tb*-containing phagosomes is arrested, and the bacteria survive within the host macrophage. *M.tb* resides within a novel vacuole that retains characteristics of early endosomal compartments and fails to fuse with lysosomes (Russell 2003; Vergne et al. 2004). Many researchers have focused on the disturbed proteins on *M.tb*-containing phagosomes to reveal the specific mechanisms of *M.tb* survival in macrophages. Early work showed that *M.tb* blocks phagosome maturation by Rab5 and Rab7, two small GTPases associated with membrane trafficking in the fusion of the phagosome and lysosome (Via et al. 1997). Later researchers identified a key step of phagosome fusion, the delivery of Cathepsin D and the H^+^-ATPase subunit Vo to the phagosome. This step is mediated by the recruitment of the Rab5 effector molecule EEA1. The recruitment of EEA1 to the membrane of the phagosome is highly dependent on the association of its FYVE domain with PI3P, which it is generated from PI by the PI3 kinase VPS34. Our data also demonstrated decreased activity of VPS34 in an *M.tb*-infected macrophage mode (Fratti et al. 2003a).

SD is a pentacyclic triterpenoid compound isolated from *A. umbellate*. In previous reports, various types of plant triterpenoids have been reported to exhibit antitubercular activity (Akihisa et al. 2005; Jyoti et al. 2015). However, few studies have focused on the intracellular inhibitory activity of triterpenoids in *M.tb* infection. In this study, we investigated the inhibitory activity of SD in *M.tb*-infected macrophages. Intracellular replication tests were performed after 4 weeks of incubation of *M.tb* with macrophage lysates. Different CFU numbers reflected different viabilities of intracellular *M.tb* in macrophages (Rampini et al. 2008). SD inhibited intracellular growth of *M.tb* 48 h post-infection as determined by the intracellular replication test. Based on results showing that *M.tb* can also infect alveolar epithelial cells, we also treated *M.tb*-infected A549 cells with SD (Castro-Garza et al. 2012).We found that SD only suppressed the intracellular replication of *M.tb* in macrophages. This observation suggested that inhibition of intracellular bacteria might depend on a phagocytosis-related mechanism. As phagosomal maturation arrest is one of the common features during *M.tb* infection, we then studied the influence of SD on phagosomal maturation arrest during *M.tb* infection. A mature phagosome has a pH of less than 5 (Rohde et al. 2007). To visualize the mature phagosome during *M.tb* infection, we treated THP-1-derived macrophages with LysoTracker, which stained the compartments with a pH below 6 (von Bargen et al. 2009). The co-localization of *M.tb* and mature phagosomes increased approximately 21% in SD-treated macrophages compared to that of the non-treated cells, indicating an increased phagocytic capacity following SD treatment.

VPS34, as a member of the phosphoinositide 3-kinase (PI3K) family of lipid kinases, is activated by forming a complex with a series of proteins. Most intracellular VPS34 forms a minimal complex, the VPS34–VPS15 dimer. Current nomenclature refers to the tetramer containing VPS34, VPS15, Beclin-1 and UVRAG (UV radiation resistance-associated gene) as complex II, which is required for the membrane fusion of phagosomes and lysosomes (Lamb et al. 2013). Over-expression of UVRAG and increased levels of the intracellular VPS34/VPS15/Beclin-1/UVRAG complex resulted in increased lipid kinase activity (Sun et al. 2011; Zhong et al. 2009). Our immunoprecipitation assays showed that SD treatment increased the proportion of VPS34/VPS15/Beclin-1/UVRAG tetramers in the VPS34 pool. AMPK signalling is an important activator upstream of both the VPS34 complex I and complex II. AMPK phosphorylates VPS34 and Beclin1 and enhances the lipid kinase activity of the VPS34 tetramer (Xu et al. 2016). Based on our assays, the increased level of AMPK phosphorylation may be the probable mechanism of activation of VPS34 tetramer induced by SD during *M.tb* infection. Therefore, SD promotes phagosomal maturation of macrophages during *M.tb* infection, and the specific mechanisms may be a pAMPK/VPS34 complex II-dependent cascade.

In this study, SD was shown to promote phagosome maturation and inhibit intracellular *M.tb* replication in THP-1-derived macrophages. The underlying mechanism was highly related to the activation of VPS34 complex II through an AMPK-dependent signalling cascade. These results suggest that a traditional Chinese herb may be used to treat infection of *M.tb*.

## Abbreviations

SD: saxifragifolin D
*M.tb*: *M. tuberculosis*
TB: tuberculosis
BCG: Bacille–Calmette–Guerin
EEA: early endosome antigen
PI3P: phosphatidylinositol 3-phosphate,1,2-dipalmitoyl
ManLAM: Mannose-capped lipoarabinomannan
UVRAG: UV radiation resistance associated
PMA: phorbol 12-myristate 13-acetate
P and B: Proskauer and Beck
MOI: multiplicity of infection
SDS-PAGE: sodium dodecyl sulphate polyacrylamide gel electrophoresis
PVDF: polyvinylidene difluoride
Co-IP: co-immunoprecipitation
DOR: dorsomorphin
PI3 K: phosphoinositide 3-kinase

## References

[CR1] Akhtar N, Syed DN, Khan MI, Adhami VM, Mirza B, Mukhtar H (2016). The pentacyclic triterpenoid, plectranthoic acid, a novel activator of AMPK induces apoptotic death in prostate cancer cells. Oncotarget.

[CR2] Akihisa T, Franzblau SG, Ukiya M, Okuda H, Zhang F, Yasukawa K, Suzuki T, Kimura Y (2005). Antitubercular activity of triterpenoids from Asteraceae flowers. Biol Pharm Bull.

[CR3] Castro-Garza J, Swords WE, Karls RK, Quinn FD (2012). Dual mechanism for *Mycobacterium tuberculosis* cytotoxicity on lung epithelial cells. Can J Microbiol.

[CR4] Chua J, Deretic V (2004). *Mycobacterium tuberculosis* reprograms waves of phosphatidylinositol 3-phosphate on phagosomal organelles. J Biol Chem.

[CR5] Ejim L, Farha MA, Falconer SB, Wildenhain J, Coombes BK, Tyers M, Brown ED, Wright GD (2011). Combinations of antibiotics and nonantibiotic drugs enhance antimicrobial efficacy. Nat Chem Biol.

[CR6] Flannagan RS, Jaumouille V, Grinstein S (2012). The cell biology of phagocytosis. Annu Rev Pathol.

[CR7] Fratti RA, Backer JM, Gruenberg J, Corvera S, Deretic V (2001). Role of phosphatidylinositol 3-kinase and Rab5 effectors in phagosomal biogenesis and mycobacterial phagosome maturation arrest. J Cell Biol.

[CR8] Fratti RA, Chua J, Deretic V (2003). Induction of p38 mitogen-activated protein kinase reduces early endosome autoantigen 1 (EEA1) recruitment to phagosomal membranes. J Biol Chem.

[CR9] Fratti RA, Chua J, Vergne I, Deretic V (2003). *Mycobacterium tuberculosis* glycosylated phosphatidylinositol causes phagosome maturation arrest. Proc Natl Acad Sci USA.

[CR10] Jyoti MA, Zerin T, Kim TH, Hwang TS, Jang WS, Nam KW, Song HY (2015). In vitro effect of ursolic acid on the inhibition of *Mycobacterium tuberculosis* and its cell wall mycolic acid. Pulm Pharmacol Ther.

[CR11] Lamb CA, Yoshimori T, Tooze SA (2013). The autophagosome: origins unknown, biogenesis complex. Nat Rev Mol Cell Biol.

[CR12] Maxmen A (2013). Ahead of WHO meeting, experts clash over tuberculosis targets. Nat Med.

[CR13] Park JH, Kwak JH, Khoo JH, Park SH, Kim DU, Ha DM, Choi SU, Kang SC, Zee OP (2010). Cytotoxic effects of triterpenoid saponins from *Androsace umbellata* against multidrug resistance (MDR) and non-MDR cells. Arch Pharm Res.

[CR14] Purdy GE, Owens RM, Bennett L, Russell DG, Butcher BA (2005). Kinetics of phosphatidylinositol-3-phosphate acquisition differ between IgG bead-containing phagosomes and *Mycobacterium tuberculosis*-containing phagosomes. Cell Microbiol.

[CR15] Rampini SK, Selchow P, Keller C, Ehlers S, Bottger EC, Sander P (2008). LspA inactivation in *Mycobacterium tuberculosis* results in attenuation without affecting phagosome maturation arrest. Microbiology.

[CR16] Rohde K, Yates RM, Purdy GE, Russell DG (2007). *Mycobacterium tuberculosis* and the environment within the phagosome. Immunol Rev.

[CR17] Russell DG (2003). Phagosomes, fatty acids and tuberculosis. Nat Cell Biol.

[CR18] Russell DG (2011). *Mycobacterium tuberculosis* and the intimate discourse of a chronic infection. Immunol Rev.

[CR19] Russell RC, Tian Y, Yuan H, Park HW, Chang YY, Kim J, Kim H, Neufeld TP, Dillin A, Guan KL (2013). ULK1 induces autophagy by phosphorylating Beclin-1 and activating VPS34 lipid kinase. Nat Cell Biol.

[CR20] Shi JM, Bai LL, Zhang DM, Yiu A, Yin ZQ, Han WL, Liu JS, Li Y, Fu DY, Ye WC (2013). *Saxifragifolin D* induces the interplay between apoptosis and autophagy in breast cancer cells through ROS-dependent endoplasmic reticulum stress. Biochem Pharmacol.

[CR21] Stokes RW, Haidl ID, Jefferies WA, Speert DP (1993). Mycobacteria-macrophage interactions. Macrophage phenotype determines the nonopsonic binding of *Mycobacterium tuberculosis* to murine macrophages. J Immunol.

[CR22] Sun Q, Zhang J, Fan W, Wong KN, Ding X, Chen S, Zhong Q (2011). The RUN domain of rubicon is important for hVps34 binding, lipid kinase inhibition, and autophagy suppression. J Biol Chem.

[CR23] Vergne I, Chua J, Singh SB, Deretic V (2004). Cell biology of *mycobacterium tuberculosis* phagosome. Annu Rev Cell Dev Biol.

[CR24] Via LE, Deretic D, Ulmer RJ, Hibler NS, Huber LA, Deretic V (1997). Arrest of mycobacterial phagosome maturation is caused by a block in vesicle fusion between stages controlled by rab5 and rab7. J Biol Chem.

[CR25] von Bargen K, Polidori M, Becken U, Huth G, Prescott JF, Haas A (2009). Rhodococcus equi virulence-associated protein A is required for diversion of phagosome biogenesis but not for cytotoxicity. Infect Immun.

[CR26] Wang Y, Zhang D, Ye W, Yin Z, Fung KP, Zhao S, Yao X (2008). Triterpenoid saponins from Androsace umbellata and their anti-proliferative activities in human hepatoma cells. Planta Med.

[CR27] Xu DQ, Wang Z, Wang CY, Zhang DY, Wan HD, Zhao ZL, Gu J, Zhang YX, Li ZG, Man KY, Pan Y, Wang ZF, Ke ZJ, Liu ZX, Liao LJ, Chen Y (2016). PAQR3 controls autophagy by integrating AMPK signaling to enhance ATG14L-associated PI3K activity. EMBO J.

[CR28] Yang CS, Kim JJ, Lee HM, Jin HS, Lee SH, Park JH, Kim SJ, Kim JM, Han YM, Lee MS, Kweon GR, Shong M, Jo EK (2014). The AMPK-PPARGC1A pathway is required for antimicrobial host defense through activation of autophagy. Autophagy.

[CR29] Zhong Y, Wang QJ, Li X, Yan Y, Backer JM, Chait BT, Heintz N, Yue Z (2009). Distinct regulation of autophagic activity by Atg14L and Rubicon associated with Beclin 1-phosphatidylinositol-3-kinase complex. Nat Cell Biol.

[CR30] Zumla A, Nahid P, Cole ST (2013). Advances in the development of new tuberculosis drugs and treatment regimens. Nat Rev Drug Discov.

